# Protocol for a cluster randomised controlled trial of a Peer-Led physical Activity iNtervention for Adolescent girls (PLAN-A)

**DOI:** 10.1186/s12889-019-7012-x

**Published:** 2019-05-28

**Authors:** Kathryn Willis, Byron Tibbitts, Simon J. Sebire, Tom Reid, Stephanie J. MacNeill, Emily Sanderson, William Hollingworth, Rebecca Kandiyali, Rona Campbell, Ruth R. Kipping, Russell Jago

**Affiliations:** 1 Centre for Exercise, Nutrition & Health Sciences, School for Policy Studies, University of Bristol, 8 Priory Road, Bristol, BS8 1TZ UK; 2 Bristol Medical School: Population Health Sciences, University of Bristol, Bristol, UK; 3 Bristol Randomised Trials Collaboration, Bristol Trials Centre, University of Bristol, Bristol, UK

**Keywords:** Physical activity, Peers, Adolescent girls, Intervention, School

## Abstract

**Background:**

Adolescent girls are less physically active than recommended for health, and levels decline further as they approach adulthood. Peers can influence adolescent girls’ physical activity. Interventions capitalising on peer support could positively impact physical activity behaviour in this group. Building on promising feasibility work, the purpose of this cluster randomised controlled trial is to assess whether the Peer-Led physical Activity iNtervention for Adolescent girls (PLAN-A) increases adolescent girls’ physical activity and is cost effective.

**Methods:**

PLAN-A is a two-arm secondary school-based cluster randomised controlled trial, conducted with girls aged 13–14 years from twenty schools in the south west of England. The intervention requires participants to nominate influential girls within their year group to become peer supporters. The top 15% of girls nominated in each school receive three days of training designed to prepare them to support their peers to be more physically active during a ten-week intervention period. Data will be collected at two time points, at baseline (T0) and 5–6 months post-intervention (T1). Schools will be randomly allocated to the intervention (*n* = 10) or control (*n* = 10) arm after T0. At each time point, all consenting participants will wear an accelerometer for seven days to assess the primary outcome of mean weekday minutes of moderate-to-vigorous physical activity. Multivariable mixed effects linear regression will be used to estimate differences in the primary outcome between the two arms and will be examined on an Intention-to-Treat (ITT) basis. A self-report psychosocial questionnaire will be completed by participants to assess self-esteem and physical activity motivation. Resource use and quality of life will be measured for the purposes of an economic evaluation. A mixed-methods process evaluation will be conducted to explore intervention fidelity, acceptability and sustainability. Analysis of quantitative process evaluation data will be descriptive, and the framework method will be used to analyse qualitative data.

**Discussion:**

This paper describes the protocol for the PLAN-A cluster randomised controlled trial, a novel approach to increasing adolescent girls’ physical activity levels through peer support.

**Trial registration:**

ISRCTN14539759–31 May, 2018.

**Electronic supplementary material:**

The online version of this article (10.1186/s12889-019-7012-x) contains supplementary material, which is available to authorized users.

## Background

Among adolescents, physical activity is associated with reduced risk of obesity and improved fitness, muscle and bone strength and mental health [1–4]. A number of studies [5, 6] report that large proportions of adolescents do not meet the Chief Medical Officer’s recommendation of an hour of moderate-to-vigorous physical activity (MVPA) per day. Physical activity levels can decrease by as much as 7% per year throughout adolescence [5, 6] with the decline starting sooner and becoming steeper for girls than for boys [6, 7]. Thus, there is a clear need to increase physical activity among adolescent girls.

Promoting young people’s health in schools is a public health priority [8] and systematic reviews have looked at the effectiveness of school-based physical activity interventions on levels of MVPA and its impact on health indicators [9–11]. A recent meta-analysis of 17 school-based physical activity interventions for girls indicated they only had a small positive effect. [11]. Similar results have been found elsewhere [12, 13]. Many of these multi-component interventions focussed on top-down strategies, and existing reviews encourage researchers to explore novel approaches to increase physical activity [10].

Factors influencing girls’ physical activity levels and participation include psychological correlates such as perceived competence, self-efficacy motivation, attitude and enjoyment of physical activity, as well as external factors such as competing priorities, friendship group changes, ‘sporty’ stereotypes, and family and peer support [14–17]. Peers play a pivotal role in adolescents’ physical activity through social support, peer presence, peer norms, the quality of friendships, peer affiliation and peer victimisation [18, 19]. Evidence also suggests that adolescents socialise in groups with similar physical activity levels and, over time, their physical activity behaviours reflect those of their peers [20]. Peer-based interventions could be an effective means of helping adolescents become more physically active [18, 21].

Several peer-based interventions have aimed to increase physical activity among adolescents. A large randomised controlled trial [22], in which older pupils mentored younger, same sex pupils using a booklet addressing barriers to physical activity and setting activity goals in weekly meetings, found no evidence of an impact on MVPA. A pilot, reward-based intervention involving the combination of older and same age peers to encourage others to try new activities, resulted in a 5.1 (95% CI = 1.1–9.2) minute difference in objectively assessed MVPA in favour of the intervention group [23].

The peer-led health intervention ASSIST (A Stop Smoking In Schools Trial) was successful in reducing the odds of 12–13 year olds being a smoker up to 2 years post intervention by 22% [24]. ASSIST utilised Diffusion of Innovations (DOI) theory [25], which suggests that social influencers amongst a group of individuals can act as change agents, using their capacity to influence change in social norms, which in turn can lead to changes in behaviour. Pupils in ASSIST were asked to nominate influential students in their year group to become ‘peer supporters’ and hold informal conversations with their peers about the risks of smoking and the benefits of being smoke-free. The process evaluation revealed that asking peer supporters to work informally, rather than under the supervision of teaching staff, meant they took the responsibility seriously and were more effective at passing on messages about not smoking to their peers [26].

The approach taken in ASSIST has been adopted in PLAN-A (Peer-Led physical Activity iNtervention for Adolescent girls), which has a theoretical underpinning using DOI together with Self-determination Theory (SDT) [27]. Within SDT, positive and sustained behaviour change and well-being is likely if motivation for physical activity is based on authentic choice and personal value, supported by an environment that fosters an individuals’ autonomy, competence, and relatedness [28, 29]. SDT is well suited to a peer-based intervention because peers can create a social climate that can undermine or facilitate girls’ interest in physical activity [30]. Previous research has shown that interventions which have a theoretical underpinning are more likely to be effective in changing adolescent girls’ physical activity [11, 31], however, few have been theory-based [30]. In contrast, numerous elements of the PLAN-A intervention, including the design, delivery and content were informed by DOI and SDT.

The PLAN-A intervention has been developed as a novel approach to increase adolescent girls’ physical activity by capitalising on the power of peer influence by promoting peer support and enhancing communication between peers. It addresses barriers to girls’ physical activity participation, seeks to create new peer-norms for physical activity whilst building on previous successful, sustainable peer-led interventions.

The aim of this cluster-randomised controlled trial is to explore whether PLAN-A is effective and cost-effective at increasing adolescent girls’ (13–14 years) physical activity. The four specific research objectives are to:

1. Determine the effectiveness of PLAN-A to increase objectively-assessed (accelerometer) mean weekday minutes of MVPA among Year 9 girls 5–6 months after the end of a 10-week intervention.
2. Determine the effectiveness of PLAN-A to improve the following secondary outcomes among Year 9 girls 5–6 months after the end of a 10-week intervention:
  - Mean weekend minutes of MVPA
  - Mean weekday minutes of sedentary time (accelerometer-derived)
  - Mean weekend minutes of sedentary time (accelerometer-derived)
  - Self-esteem (self-reported [32])
3. Determine the extent to which any effects of the intervention on primary or secondary outcomes are mediated by autonomous and controlled motivation towards physical activity and perceptions of autonomy, competence and relatedness / peer support in physical activity.
4. Determine the cost-effectiveness of PLAN-A from a public sector perspective.

## Methods/design

### Study design

**Fig. 1:**
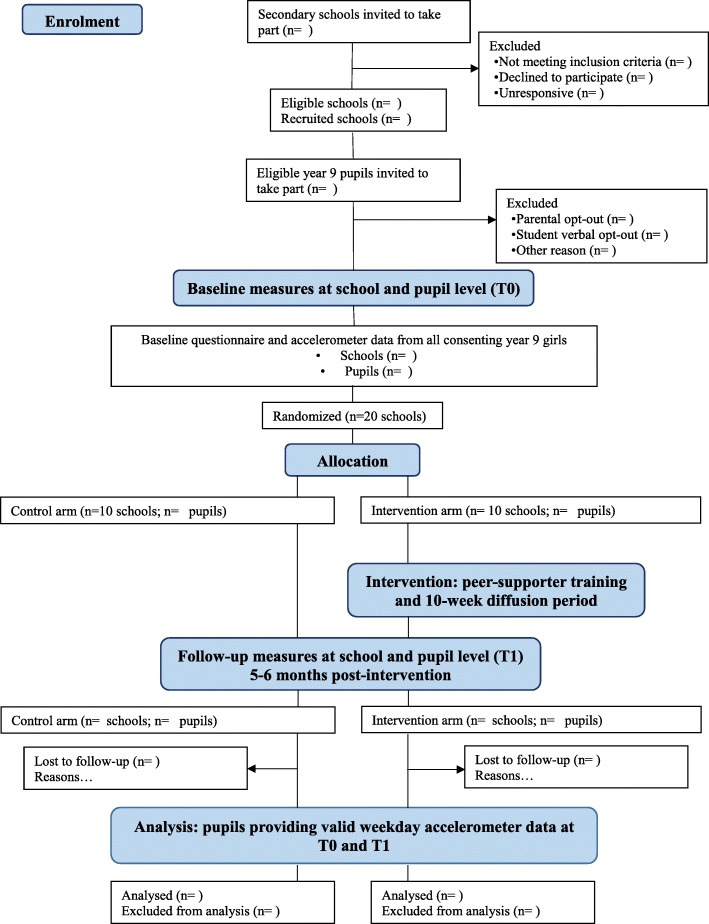
PLAN-A study flow diagram

PLAN-A is a two-arm school-basedcluster-randomised controlled trial. Schools will be the unit of randomisation and outcomes will be assessed at two time points: baseline (Time 0: Autumn term of Year 9) and follow-up (Time 1: Autumn term of Year 10, 5–6 months post-intervention). Twenty schools will be randomly allocated after completion of baseline data collection using a 1:1 allocation. Figure 1 shows the study flow diagram.

A comprehensive mixed-methods process evaluation will be conducted, together with an economic evaluation to estimate cost-effectiveness at Time 1 and extrapolate beyond the end of the trial. The SPIRIT diagram (Table 1) provides an overview of the scheduling of the intervention and research elements, the details of which are described below. The SPIRIT checklist, listing the recommended items to address in a trial protocol, can be found in Additional file 1.

**Table 1:** PLAN-A SPIRIT diagram displaying study recruitment, intervention and measures schedule

	STUDY PERIOD			
	Enrolment	Allocation	Post-allocation	Follow up/close out
TIMEPOINT	*T0*	*Randomization*	*Intervention*	*T1*
ENROLMENT:				
*Informed consent*	X			
*Baseline measures*	X			
*Randomization into study arm*		X		
INTERVENTIONS:				
*Peer nomination*	X			
*Trainer recruitment and training*	X			
*Peer-supporter training (10 schools)*			X	
*Normal treatment (10 schools)*			X	
ASSESSMENTS:				
*Percentage opt-in (total, by sex)*	X			
*Retention/loss to follow up*				X
*Participant characteristics*	X			X
*Self-reported psychosocial questionnaires*	X			X
*Health related quality of life questionnaires (EQ-5D-Y, KIDSCREEN-10)*	X			X
*Resource use proformas (peer nomination and school contact)*	X		X	
*Mean daily moderate to vigorous physical activity*	X			X
*Process Evaluation*			X	X

### Setting and participants

The study setting will be state-funded secondary schools in the South West of England. All schools, apart from special educational and independent schools, will be eligible to participate. However, the opportunity to participate will be given first to schools above the median of the local Pupil Premium Indicator (i.e. more deprived). If this method does not meet the recruitment target, all other schools will be invited. The target population is girls in Year 9 (aged 13–14 years) attending eligible schools. All female Year 9 pupils will be eligible to participate and, in intervention schools, will be targeted in the intervention. A subgroup (≥15%) of Year 9 girls in each intervention school will be trained as peer supporters.

### Recruitment

#### School recruitment

Schools meeting the inclusion criteria will be invited to take part with a letter to the Head or Deputy Head Teacher and meetings with the project team. We will aim to recruit two reserve schools who will enter the study if a participating school chooses to withdraw prior to baseline data collection.

#### Pupil recruitment

Year 9 girls will be recruited to participate in the study via a presentation from the study team in school informing them about the trial, the randomisation process and the intervention. All girls will be invited to take part and given an information pack for themselves and their parents. We will employ a dual parent and pupil consent process. Girls eligible to participate must provide written informed consent. Parents can opt their daughter out of the study by providing written informed opt-out. These girls, or those who do not provide consent, will be excluded from the study. Peer supporters will be asked to consent to the role and focus groups. Parents will be asked to give consent to allow their daughter to attend the peer-supporter training. If girls have been nominated as a peer supporter, but have not consented to the data collection process, they will still be eligible to participate in the project as a peer supporter. Adult participants (e.g., peer-supporter trainers & school contacts) will provide written informed consent. At all time points pupils will be able to withdraw should they wish. This study was approved by the School for Policy Studies Ethics and Research Committee at the University of Bristol (REF: SPSREC17–18.C22) on 30/05/2018.

#### Trainer recruitment

Trainers to deliver the peer-supporter training will be employed as free-lancers. In keeping with a public sector approach, the opportunity to become a PLAN-A trainer will be advertised via Local Authority health improvement teams.

### Allocation

School is the unit of allocation. Twenty schools will be randomly allocated after completion of baseline measures; ten intervention and ten control schools. Randomisation will be stratified by county (Avon, Devon and Wiltshire) and the England IMD score for the local super output area where the school is located to ensure balance within each stratum. The latter will be dichotomised as either above or below the median of sampled schools in the county. Random allocation will be performed (computer generated) by a member of the Bristol Randomised Trials Collaboration (BRTC: a UKCRC-registered Clinical Trials Unit) who will be blind to school identity and independent of the fieldwork team.

### Sample size

The PLAN-A feasibility study [33] found a between-arm difference in mean weekday MVPA of 6.1 min with 95% confidence intervals between 1.4 to 10.8 min, suggesting that a target between-arm difference of 10 min of MVPA per weekday is achievable. Recognising that even smaller intervention effects on MVPA may also lead to meaningful differences in health at a population level, the sample size necessary to detect a range of differences in weekday MVPA (i.e., 10, 8 & 6 min) were calculated. Table 2 shows the power calculations where the following parameters are fixed: cluster size = 70 (informed by feasibility study [33]), intra class correlation (ICC) on weekday MVPA = 0.01 (informed by the feasibility study; T0 = <.0, T1 = .02, T2 = <.0001 and other studies [34, 35]), MVPA standard deviation = 20 min (based on feasibility study), coefficient of variation in cluster size of 0.22, 5% two-sided alpha and inflation to account for 30% of participants not providing primary outcome data. 12 schools and 800 pupils are required to detect a 10-min difference in MVPA with 90% power, however 20 schools and 1400 pupils would provide 90% power to detect a smaller 6-min difference in MVPA and ample power to detect an 8-min difference. Further accounting for correlation between baseline and follow-up measures of MVPA (ρ = 0.4) slightly reduces the number of schools required (last column, Table 2).

**Table 2:** Sample size parameters

MVPA Difference (mins)	Power	N pupils (uninflated)	N pupils (inflated)	N Schools	N Schools when allowing for correlation between baseline and follow-up
10	90	560	800	12	10
10	80	420	600	10	10
8	90	700	1000	16	12
8	80	560	800	12	12
6	90	980	1400	20*	18
6	80	840	1200	18	16

*sample size used

Given the inherent uncertainty in many of these assumptions, we will recruit 22 schools (20 + 2 reserves) and randomise 20 schools and 1400 pupils to detect a 6-min difference in MVPA with at least 90% power.

### The PLAN-A intervention

The intervention design was adapted from the intervention model used in the ASSIST trial [24] to focus on increasing adolescent girls’ physical activity. The intervention consists of different elements:

A) *Peer nomination:* All Year 9 girls will be asked to complete a peer nomination form asking them to identify influential peers within their year (e.g. who they respect, trust, listen and look-up to). The highest scoring 18% [25] (with the aim of ≥15% providing consent) of girls in intervention schools will be invited to take on the role of a peer supporter at a meeting with the study team, where they will be given written information for themselves and their parents.
B) *Train-the-trainers:*Peer-supporter training (described below) will be delivered by free-lance female trainers selected for their existing experience and physical activity subject knowledge. Trainers will receive 3 days training which cover the PLAN-A design and concept, role-play delivery of each activity, how to deal with challenging behaviour, the principles behind DOI and SDT and how the trainers can deliver the training in a style to increase peer supporter autonomy (e.g., empowerment to support peers and provide choice), competence (e.g., in how to be a peer-supporter) and belonging (e.g., supportive network of peer-supporters). This training will be co-delivered by an experienced PLAN-A trainer from the feasibility study alongside a member of the study team in order to more closely model how training would be delivered if the intervention was rolled out in the community.
  All trainers will be issued resources to help facilitate the delivery of the peer-supporter training. These include a ‘Trainers’ guide’ with information about PLAN-A, the underpinning theoretical principles driving design of the content and delivery systems, the role of a PLAN-A trainer, activities and training logistics, and a ‘Session plans’ booklet detailing how to deliver each activity of the peer-supporter training. A resources pack will be supplied for each peer-supporter training session containing activity materials and games for break times.
C) *Peer-supporter training*: Peer supporters will attend an initial two-day training course to develop the skills, knowledge and confidence to promote physical activity amongst their close peers. A one-daytop-up training will be held mid-way through the 10-week diffusion period, the purpose being to revisit core topics, share successes and resolve problems. Each peer supporter will receive a combined ‘Peer supporter booklet and diary’ to support the content of the training. The booklet contains information and worksheets that are part of the training, and a diary is provided to peer supporters to add details about conversations they have with their peers. Appropriate sites near to school (e.g. leisure centres, community halls), but away from the normal school environment, will be used for the training. The peer-supporter training has been informed by formative and feasibility research and is designed to be mentally and physically engaging. It addresses issues central to girls’ physical activity including health benefits, active choices, developing an active identity, being active with friends, sedentary behaviour, communicating with confidence, empathy and supporting motivation. As well as being framed by DOI to capitalise on peer influence potential, training content is grounded in SDT to build the girls’ perceived autonomy, competence and social support for being a peer supporter in relation to physical activity and when supporting their peers. Specifically, resources and training content are designed to encourage peer supporters to recognise and promote autonomous rather than controlled motivation for physical activity (focussing on health, challenge-seeking & social reasons rather than appearance & peer pressure).
D) *10-week intervention:* On completion of the training, peer supporters will be encouraged to informally promote physical activity amongst their close peers for 10 weeks (with the top-up training day at 5 weeks). The foundation of the intervention is an informal peer-led approach; therefore, girls can choose how they wish to support specific friends or groups based on their knowledge of them, their preferences, needs, confidence etc., which the peer-supporter training helps girls to identify and respond to with empathy. However, ideas and techniques on how to encourage and support their peers to be active are also provided at the peer-supporter training. These include having conversations, co-participation, persuading and offering support or encouragement.

### Control group provision

All consenting Year 9 pupils in control schools will participate in T0 and T1 data collection, including peer nomination, however the 10 schools assigned to the control condition will not receive the intervention and will continue with normal practice. Results of the peer nomination will be made known to the control schools after T1 data collection.

### Data collection

All primary and secondary measures will be taken at baseline (T0) and at 5–6 months post intervention (T1). At T0 only, participants will be asked the following descriptive variables: 1) home postcode to derive Index of Multiple Deprivation (IMD) estimates, 2) ethnicity, 3) family affluence [36], 4) whether they receive free school meals. The primary outcome - objective minutes of weekday MVPA, will be assessed using ActiGraph wGT3X+ and wGT3X-BT accelerometers. Participants will be asked to wear the devices for seven consecutive days at T0 and T1. Periods of ≥60 min of zero counts will be classified as ‘non-wear’ and removed. Participants will be included in the primary outcome analysis if they provide ≥2 valid weekdays of data (500 min of data between 06:00 and midnight). Evenson [37] cut points have been found to be the most accurate MVPA threshold for adolescents [38] and will be used., We will also estimate participants’ sedentary time using a cut-point of less than 100 counts per minute [37]. The following secondary outcomes will also be assessed using an ActiGraph accelerometer, mean weekend minutes of MVPA, and mean weekday and weekend minutes of sedentary time.

At both time points, participants will report their self-esteem using the Self-Description Questionnaire [32], and the three central constructs of SDT (autonomy [39], competence [40] and relatedness [39] need satisfaction) and self-determined physical activity motivation (Behavioural Regulation in Exercise Questionnaire-2, BREQ-2) [41] will be assessed using validated self-report questionnaires. Physical activity social support will also be assessed at T0 and T1 using two questions designed in the pilot study specifically for PLAN-A: 1) Has anyone in your year group talked with you recently about physical activity? (Yes, no I’m not sure) and 2) Did talking to anyone in your year help you to be more active? (Yes, no, I’m not sure, I didn’t speak to anyone).

#### Process evaluation

The purpose of the mixed methods process evaluation will be to examine a) intervention implementation and fidelity, b) intervention receipt (school, pupil and peer-supporters) and c) sustainability. Table 3 describes the methods that will be used in the process evaluation, by informant group.

**Table 3:** Process evaluation methods

Informant	Method	Data collected	Time point
Peer supporter (PS)	Register	Training attendance	PS training days
	Questionnaire	Quantitative and qualitative feedback about training enjoyment, activities, ability to prepare girls to peer support and trainer autonomy support	Post two-day and top-up PS training
	Questionnaire	Quantitative and qualitative feedback regarding the types, frequency and extent of support given to peers	Week 5 and 10 of intervention
	Focus groups	Thoughts on the PS training (and trainers), on their role as a peer supporter (enjoyment, successes, challenges, impact), and PS actions	Post intervention
Trainer	Questionnaire	Quantitative ratings about the PS training arrangements, objective fulfilment and PS engagement	Post two-day and top-up PS training
	Training observation	Observations on PS training logistics, PS engagement, intervention fidelity and trainer style	PS training days
	Semi-structured interviews	Feedback about the train-the-trainers, delivery of PS training and improvements	Post top-up PS training
Non-peer supporter	Questionnaire	Intervention schools only – contact with PS and its perceived impact	T1
	Focus groups	Awareness of PLAN-A, thoughts on the PS, and perceived impact (did they talk to a PS? If so, did it help them change their behaviour?)	Post intervention
School contact	Semi-structured interview	Level of involvement with the study, data collection arrangements, intervention implementation, potential impact and sustainability	Post intervention
Public health commissioners	Semi-structured interview	Advice about sustainability and dissemination, funding and roll-out	Post intervention

An audit will be conducted to assess school context. The tool, adapted and tested in the PLAN-A feasibility trial, will evaluate the quantity and quality of equipment/facilities in the school that promote physical activity among its pupils [42], as well as the schools’ physical activity policies and physical activity throughout the curriculum [43]. In addition, data on school size, pupil premium and termly after-school provision will be collected in order to account for school-specific factors that could explain changes in MVPA observed at T1. Any adverse events or unintended effects will be recorded and reported to the Chair of the Trial Steering Committee and the Chair of the ethics committee.

#### Economic data

Resource use will be collected on all aspects of intervention set-up and delivery. These include research staff, trainer, school staff and pupil time, expenses, travel, materials, venue hire costs and administration. Time burden for each component of intervention refinement and delivery will be logged by the research team, with respect to staff grade to determine cost. School staff and pupil time for peer nomination and intervention delivery will be recorded on proforma by research staff after consultation with school contacts. Intervention resources such as printed manuals, worksheets and equipment to facilitate the delivery of activities will be itemised, and the cost of hiring and transporting pupils to venues for the training will be recorded per school. Although the intervention has the potential to influence participants healthcare use long-term, we will not collect this information within the trial as we wish to minimise participant burden and do not believe the intervention is likely to influence healthcare use in the short term. Participant health-related quality of life will be measured using the EQ-5D-Y [44] and KIDSCREEN-10 [45] questionnaires.

### Participant appreciation

All participating schools will receive a £500 donation and a summary of project findings as appreciation for devoting time to the project. All consenting girls will receive a £10 ‘Love to Shop’ voucher after completing measures at each time point (£20 in total) to recognise their contribution to the project.

### Data analysis

Data will be entered electronically on a secure file store system and password protected. Data will be anonymised by assigning a unique identification number to each pupil.

#### Quantitative analysis

Trial outcome data will be reported in line with Consolidated Standards of Reporting Trials (CONSORT) guidance [46] and intervention elements will be described using the Template for Intervention Description and Replication (TIDieR) checklist [47]. The primary comparative analysis will be examined on an Intention-to-Treat (ITT) basis including all participants in randomisation without imputation for missing data. Multivariable mixed effects linear regression will be used to estimate differences in the primary outcome; objectively-assessed mean weekday minutes of MVPA, between intervention and control groups adjusting for baseline outcome score and randomisation variables. Secondary analysis will be similar and will adjust for further imbalanced variables between trial arms at baseline. Similar analyses will be repeated for secondary outcomes. A sensitivity analysis, using a suitable imputation method, will be conducted to assess the effect of missing data. *P*-values and 95% confidence intervals will be calculated. A small number of pre-specified subgroup analyses will be carried out to evaluate whether the intervention is differentially effective/cost-effective in different subgroups, such as by school-level socioeconomic position. The trial is not powered to detect effectiveness in subgroups, and this analysis will be treated as exploratory, presented using confidence intervals and interpreted with caution. Mediation analysis [48] will look at whether any intervention effect is mediated by the SDT constructs; autonomous motivation, perceived competence and peer norms for physical activity. No secondary per-protocol analysis is planned due to the informal nature of the intervention. However, if a school fails to deliver the intervention, a per-protocol analysis will be conducted based on whether the intervention training was delivered (yes/no). An additional sensitivity analysis of the intervention effect on the primary outcome will be performed if the school context audit shows a between arm difference of new school physical activity provision.

A public sector perspective will be taken in the economic analysis, including costs to Local Authorities and schools. To increase generalisability, national unit costs for trainer and teacher time will be used where available. Time spent by peer supporters receiving training will be reported, but the opportunity cost of pupil training and dissemination will not be included in the cost-effectiveness analysis. Cost per student within each school will be estimated by dividing the costs of the peer-supporter programme at that school by the total number of female students completing the primary outcome at T1. In line with the analysis of the primary outcome, imputation will be used as a sensitivity analysis. An incremental cost effectiveness ratio (ICER) will be determined by dividing the cost per pupil of the intervention by the difference in daily MVPA in the intervention and control arms. This will be repeated in pre-specified subgroup analysis (i.e. school level socioeconomic position). EQ-5D-Y and KIDSCREEN-10 responses will be used in secondary analyses to explore whether the intervention has any short-term impact on health-related quality of life. Currently, there is no value set for the EQ-5D-Y [49], so comparison between arms will be based on raw responses to each of the five items. A mapping algorithm will be used to estimate utility scores from the KIDSCREEN-10 and compare them between arms [50]. Health economics data will be reported as outlined in the Consolidated Health Economic Evaluation Reporting Standards (CHEERS) statement checklist [51].

If there is evidence that the intervention increases MVPA, we will explore whether existing epidemiological models can be used to extrapolate how sustained increases in MVPA might affect health outcomes and healthcare utilisation in adulthood. The identification of suitable models and a pre-specified effectiveness threshold at which extrapolation would be explored will be agreed with the Trial Steering Committee and detailed in a health economics analysis plan.

#### Qualitative analysis

Semi-structured interviews and focus groups will be transcribed verbatim and anonymised before being coded. Thematic analysis techniques will be used to generate initial codes using NVivo 11 (QSR International Pty Ltd). These will be grouped to form themes for each stakeholder group. Data from different stakeholder groups will be triangulated using the Framework method [52], resulting in a matrix in which data are described by themes and codes, allowing comparisons between and within stakeholder groups to be made. To ensure we have a thorough understanding of the different perspectives and mechanisms of impact of the intervention, transcripts will be analysed using both an inductive and deductive approach with our research questions in mind. Consistency and agreement of coding will be ensured by double coding transcripts, and new or disputed codes will be discussed. These codes will be refined to create emergent themes and a framework of agreed codes will be applied to the remaining transcripts. Qualitative data will be presented in line with the Consolidated Criteria for Reporting Qualitative Research (COREQ) checklist [53].

## Discussion

This paper describes the protocol for a cluster-randomised trial of the refined PLAN-A intervention, developed as a novel approach to increasing adolescent girls’ physical activity by capitalising on the power of peer influence by promoting peer support and enhancing communication between peers. Many adolescent girls do not engage in a enough physical activity, especially within the school curriculum. Multi-component interventions have attempted to increase girls’ physical activity levels, however many of these focussed on top-down strategies which yielded little improvement. Underpinned by a combination of DOI and SDT, the PLAN-A intervention addresses barriers specific to girls’ physical activity participation, whilst building on previous successful, sustainable peer-led interventions. The goal of this study is to explore whether the PLAN-A intervention can increase adolescent girls’ (13–14 years) physical activity levels and be cost-effective from a public sector perspective.

## Additional file

Additional file 1: Spirit 2013 checklist. Recommended items to address in a clinical trial protocol and related documents. (DOC 125 kb)

## Data Availability

Not applicable

## Abbreviations

ASSIST: A Stop Smoking In Schools Trial
BREQ-2: Behavioural Regulation in Exercise Questionnaire-2
BRTC: Bristol Randomised Trials Collaboration
CHEERS: Consolidated Health Economic Evaluation Reporting Standards
CONSORT: Consolidated Standards of Reporting Trials
COREQ: Consolidated Criteria for Reporting Qualitative Research
DOI: Diffusion of Innovations
ICER: Incremental Cost Effectiveness Ratio
IMD: Index of Multiple Deprivation
ITT: Intention to Treat
MVPA: Moderate-to-vigorous physical activity
PLAN-A: Peer-Led physical Activity iNtervention for Adolescent girls
PS: Peer supporter
SDT: Self-Determination Theory
TIDieR: Template for Intervention Description and Replication
